# Summary of the 2010 AHPBA/SSO/SSAT Consensus Conference on HCC

**DOI:** 10.4061/2011/565060

**Published:** 2011-05-17

**Authors:** Gitonga Munene, Jean-Nicolas Vauthey, Elijah Dixon

**Affiliations:** ^1^Division of General Surgery, University of Calgary, AB, Canada T2N 1N4; ^2^Department of Surgical Oncology, The University of Texas, MD Anderson Cancer Center, Houston, TX 77030, USA; ^3^Department of Surgery, Foothills Medical Centre, 1403-29th Street NW, Calgary, AB, Canada T2N 2T9

## Abstract

Under the auspices of the American Hepato-Pancreato-Biliary Association, an expert consensus conference was convened in January 2010 on the multidisciplinary management of hepatocellular carcinoma. The goals of the conference were to address knowledge gaps in the optimal preparation of patients with HCC for operative therapy, best methods to control HCC while awaiting liver transplantation, and developing a multidisciplinary approach to these patients with implementation of novel systemic therapies.

## 1. Introduction

HCC has emerged as the 5th most common cancer in the world and its incidence is increasing in the Western world [1, 2] In January 2010, the American Hepato-Pancreato-Biliary Association (AHPBA) convened a consensus conference on the multidisciplinary management of hepatocellular cancer (HCC) cosponsored by the Society of Surgical Oncology, the Society for surgery of the Alimentary Tract and the The University of Texas MD Anderson Cancer center [3]. The methods used in the consensus conference have been previously described. Briefly consultation within the three sponsoring organizations identified experts to participate in the conference. Each expert was asked to present on a given area and to outline two or three consensus statements, which were then reviewed by a panel of content experts and the audience. After the symposium, the consensus statements were summarized by the speakers and session cochairs with input from the corresponding session cochairs. The meeting was divided into three sessions (1) pretreatment assessment, (2) surgical treatment, and (3) combined modality therapy [3]. The following paper provides a concise summary of the expert consensus statements resulting from the three sessions.

## 2. Pretreatment Assessment of Hepatocellular Carcinoma

Currently, there are 18 HCC scoring or staging systems used in the world, but based on current knowledge and experience no single staging system is applicable to all patients [4]. Staging systems used should combine extent of liver disease, general health, and tumor markers as features to provide guidance in prognosis and treatment. However, the use of regional staging systems should be discouraged because it precludes comparison between centers. Most staging systems studied perform poorly when used in patients with a wide spectrum of disease, and the discriminatory performance of different staging systems appears to be treatment, region, and stage specific. Given these limitations, the expert consensus was that the Barcelona Clinic liver cancer (BCLC) is appropriate for patients with advanced liver disease who are not candidates for resection and/or transplantation. BCLC also provides a reasonable guide for patients in stages B and C with the caveat that resection may be considered for some of these patients. The AJCC/UICC classification is valid in the West and East for patients undergoing liver resection, and should be coupled with the fibrosis score. Pathological outcome should be reported using the AJCC/UICC system following resection or liver transplantation. Finally accurate staging varies based on the modalities used, and optimal staging guidelines that may include biomarkers should be established to allow for more precise comparisons between different treatment regimens [4]. 

### 2.1. Pretreatment Imaging

Imaging is an integral component of pretreatment assessment of HCC and severity of liver disease. Recommendations regarding imaging were that both Dual CT and MRI should be used for pretreatment staging in HCC; however, MRI has the best performance characteristics for the detection of HCC. The use of Dual CT is also limited by repeated radiation exposure due to the frequency and length of follow-up imaging required in the management of patients with HCC and cirrhosis. Ultrasound or contrast-enhanced ultrasound could be useful for HCC screening; however, the data was insufficient to make a recommendation. Both MRI and CT have limited sensitivity and specificity for detection of lesions <1 cm; however, the new liver MR liver-specific agents are promising for HCC detection and characterization of small lesions. Image subtraction and diffusion weighted imaging should be used as markers of treatment efficacy rather than lesion size. Background liver fibrosis and cirrhosis may be also assessed by functional MRI which utilizes hepatocyte-specific contrast medium [4–6].

### 2.2. Role of Portal Vein Embolization

Portal vein embolization (PVE) has emerged as an important technique of increasing FLR (future liver remnant) in patients undergoing major hepatic resections [7–10]. The consensus regarding PVE was that patients with potentially resectable disease should have volumetric analysis of the total liver volume (TLV) and the anticipated FLR. If major hepatic resection is indicated, portal vein embolization may be appropriate when FLR < 20% of TLV in normal liver, <30% of TLV in chemotherapy associated injured liver, and <40% of TLV in patients with cirrhosis. Imaging is indicated 3-4 weeks after PVE and resection is safe when FLR volume reaches the target. Combination transarterial chemoembolization (TACE) with lipiodol and an anticancer agent followed by PVE should be considered for patients with chronic liver disease being considered for major resection due to increased hypertrophy and higher tumor responses compared to PVE alone [4, 11].

### 2.3. Defining Criteria for Resectability

The definition for resectability in HCC broadly includes two main considerations: liver function and tumor characteristics. The MELD score is useful in determining patients who can safely undergo major hepatic resection [12]. Minor resection in Child-Pugh class A patients with portal hypertension, ascites, bilirubin > 2 mg/dL is contraindicated. Resection should be considered in patients without portal hypertension and bilirubin < 1 mg/dL. Utilizing strict tumor size to determine resectability was found to be unwarranted. Multifocal tumors should be considered for resection, whereas multinodular tumors meeting the Milan criteria should be considered for transplantation given the high recurrence rates [4].

## 3. Surgical Treatment of HCC

Surgical management of HCC involves both nonresectional ablative techniques and surgical resection. Nonresectional ablative therapies have emerged as effective treatment options for patients with HCC with radiofrequency ablation (RFA) being the most commonly used technique. Percutaneous RFA has been found to induce significant tumor necrosis in small tumors away from vascular structures. Additionally, long-term survival rates after RFA are comparable to resection or liver transplant, (OLT) in patients with small HCC <2 cm [13, 14]. However this assertion is still to be determined in large randomized trials. Therefore, RFA is not recommended in resectable patients with tumors >4 cm or in HCC close to major vascular structure, but may be considered for small tumors away from vascular structures. Newer ablative therapies such as microwave ablation may be more effective in treating larger tumors and tumors close to vessels. However, current data regarding this microwave ablation and other ablative techniques such as high-intensity focused ultrasound and electropolation is immature and therefore definitive conclusions are not possible [15].

Hepatic resection is the primary treatment for HCC in selected patients with reported 5yr overall survivals of 25%–50% [16]. Selection for resection is based on the extent of the tumor and the severity of liver disease. Multiple tumors and/or portal hypertension in patients with Child-Pugh class A liver dysfunction can undergo resection with acceptable outcomes [17]. Resection with wide margins (1-2 cm) is the treatment of choice for HCC in patients without cirrhosis or for selected patients with cirrhosis without portal hypertension [16, 18]. Minimizing blood loss and performing limited resections is associated with better perioperative outcome, with most centers reporting mortality rates <5% [19]. The efficacy of resection in patients with large tumors and major vascular invasion is unclear, and decisions for surgical therapy in this group of patients must be made on an individual basis [15, 20]. Laparoscopic liver resection has been found to be feasible without compromising oncological outcome in limited clinical reports [21, 22]. 

Liver transplantation is the optimal treatment for HCC patients meeting the Milan criteria with cirrhosis where the 5 yr overall survival ranges from 60% to 80% with excellent disease-free survival [23]. However, given the limitations in available organs, the dropout rate, and the economic impact of OLT, other alternatives such as resection with equivalent outcomes should be considered in appropriate patients. OLT in patients exceeding the Milan criteria should be considered on a selective basis given the excellent outcomes observed by centers using an extended criterion [24]. Patients beyond the Milan criteria may be downstaged using locoregional therapies. Following a period of observation after downstaging, patients who meet Milan criteria may be considered for OLT [15]. 

Bridge therapies are often used to prevent progression of HCC while on the transplant list. The specific aims are: (1) avoid drop out due to HCC progression, (2) increase tumor-free survival after OLT, (3) down stage advanced HCC to enable liver transplantation, and (4) avoid delay of OLT after favorable response [25]. The common therapies utilized are RFA, TACE, percutaneous ethanol injections (PEI) and liver resection [25–29]. TACE and RFA should be considered to bridge patients due to the low morbidity and the favorable responses associated with these techniques that may reduce drop out in patients with an expected wait period of greater than 6 month prior to OLT. Liver resection should also be considered for appropriate patients where it may delay and/or avoid the need for OLT [15].

## 4. Nonoperative Therapies for Combined Modality Treatment of HCC

Most patients with HCC present with advanced liver disease and are therefore not candidates for liver transplantation, resection, or ablative procedures. However, most patients may benefit from palliative procedures that include TACE, transarterial radioembolization (TARE), external beam radiotherapy, and systemic therapy with sorafenib. Patient selection for any of these therapies is based on patient and tumor factors and decisions regarding treatment approaches should be made in a multidisciplinary setting that includes a hepatologist, interventional radiologist, and a surgeon [30].

TACE has been shown in randomized trials to increase time to progression and overall survival in patients with unresectable HCC compared to best supportive therapy or transarterial embolization [31, 32]. Based on this, TACE is a standard for intermediate-*∖*advanced-stage unresectable HCC even in the setting of portal vein thrombosis (excluding main portal vein) where there is a proven survival benefit. It is also useful in predicting tumor biology in the pretransplant setting when used for bridging or downstaging patients. Emerging data regarding the use of drug eluting microspheres TACE are encouraging due to the comparable efficacy with TACE and the potential for decreased toxicity [30].

Sorafenib which is an anti-VEGF receptor and raf kinase inhibitor is approved for the treatment of unresectable HCC and is the standard agent for systemic therapy of advanced HCC based on a level 1 data [33]. Radiographic responses to sorafenib are a poor parameter to determine response to therapy. Tumor necrosis as determined by triphasic CT may be an accurate surrogate marker of efficacy but further data is required. The extent of cirrhosis appears to influence the outcomes of sorafenib therapy. Newer novel agents require further study before recommendations can be made regarding their use [30].

The use of yttrium 90 radioembolization is safe and efficacious in well-selected groups of patient where acceptable response rates and improvements in overall survival have been reported [34]. The subsets of patients where this modality should be considered are patients being downstaged or bridged with the intention of OLT, patients with malignant portal vein thrombosis where both TACE and OLT are contraindicated, and patients with advanced disease [30, 35, 36].

Recently, there has been a resurgent interest in the use of radiotherapy for HCC, driven by technological advances and an improved understanding of hepatic tolerance to radiotherapy. External beam radiation therapy and photon irradiation have been shown to induce acceptable response rates and provide local control to unresectable tumors [37]. With improved understanding of hepatic tolerance rates, radiotherapy will further expand the treatment options for patients with HCC, and multimodal strategies that include radiotherapy merit further study [30].
